# Species differences in the morphology of transverse tubule openings in cardiomyocytes

**DOI:** 10.1093/europace/euy245

**Published:** 2018-11-23

**Authors:** Eva Alicja Rog-Zielinska, Cherrie Hei Ting Kong, Callum Michael Zgierski-Johnston, Paul Verkade, Judith Mantell, Mark Bryden Cannell, Peter Kohl

**Affiliations:** 1 Institute for Experimental Cardiovascular Medicine, University Heart Center, Faculty of Medicine, University of Freiburg, Elsässerstraβe 2Q, Freiburg, Germany; 2 School of Physiology, Pharmacology & Neuroscience, Faculty of Biomedical Sciences, University of Bristol, Bristol, UK; 3 School of Biochemistry and Wolfson Bioimaging Facility, Faculty of Biomedical Sciences, University of Bristol, Bristol, UK

**Keywords:** Heart, Cardiac, Ventricular myocyte, Ultrastructure, Electron tomography, Species differences

## Abstract

**Aims:**

The ultrastructure of ventricular cardiomyocyte T-tubule connections with the outer cell surface (‘mouth’ regions) has been reported to differ between mice and rabbits. In mice, T-tubule mouths form convoluted narrow spaces filled with electron-dense matter that impedes diffusion between T-tubular lumen and bulk extracellular space. Here, we explore whether T-tubule mouths are also constricted in rat (another murine model used frequently for cardiac research) and whether pig and human T-tubule mouth configurations are structurally more similar to mice or rabbits.

**Methods and results:**

We used chemically-fixed tissue and high-pressure frozen isolated cardiomyocytes to compare T-tubule mouth architecture using transmission electron microscopy and three-dimensional electron tomography. We find that rat T-tubular mouth architecture is more similar to that of rabbits than mice, lacking the marked tortuosity and electron-dense ground substance that obstructs access to deeper portions of the T-tubular system in mice. Pilot observations in larger mammals (pig, human) suggest that mouse may be the least representative animal model of T-tubule connectivity with the outer cell surface in larger mammals.

**Conclusion:**

Rat T-tubular system architecture appears to be more similar in size and topology to larger mammals than mice. T-tubular mouth topology may contribute to differences in experimental model behaviour, underscoring the challenge of appropriate model selection for research into cell and tissue function.

**What’s new?**

- Using electron microscopy and three-dimensional electron tomography, we show marked differences in the structure of T-tubule openings (‘mouths’) of mouse cardiomyocytes compared with rat, rabbit, pig, and human.
- Rat, pig, and human T-tubule mouths appear unobstructed and thus more structurally consistent with rabbit, while mouse T-tubule mouth regions are narrow and convoluted, containing substantial amounts of electron dense material that may impede solute access.
- The ultrastructure of T-tubule mouths should be taken into account when choosing an experimental model to study cardiac cell function, and when interpreting functional data that may depend on the configuration and structural properties of T-tubule access.

## Introduction

The uniformity of individual cardiomyocyte contraction depends on the close coupling between plasma membrane depolarization, ‘trigger’ Ca^2+^-entry, and Ca^2+^-release from the sarcoplasmic reticulum (SR).^1^ In mammalian cardiomyocytes, this is supported by the presence of an extensive T-tubular system—a complex network of surface membrane invaginations that traverse the entire cell. The topology of the cardiac T-tubular system varies between cardiac chambers: it is dominated by axial elements in atrial myocytes,^2^ while ventricular cells are dominated by transverse tubular elements. The ventricular T-tubular system varies between species, from complex meshes in rodents^3^ to predominantly radial spokes in lagomorphs.^4^ At the level of light microscopy, human T-tubular architecture appears to be closer to that of rabbit.^5^ Additionally, there are marked differences in T-tubule diameter between species, ranging from means of ∼250 nm in rats and mice,^3^^,^^6^ to ∼400 nm in rabbit and larger mammals.^6^^,^^7^ The varying geometries of the T-tubular system may contribute to differences in excitation-contraction coupling dynamics between species.

Although T-tubules are topologically situated inside the cellular silhouette, T-tubular lumina are continuous with the extracellular space, connected *via* so called ‘mouth’ regions.^3^^,^^8^ In many schematic depictions of T-tubules, the mouth areas are shown as megaphone-like cones that widen and merge with the surface sarcolemma as one progresses outwards from the associated T-tubule proper (i.e. T-tubules that form triads with neighbouring cisternae of the SR). In reality, these are dynamic structures, whose width changes during the mechanical cycle, turning from outwardly-widening cones during tissue stretch to narrow straights upon contraction.^9^ All solutes (e.g. ions, hormones, etc.) that diffuse into and out of T-tubules must traverse this mouth area, and it has been suggested that sarcomere length-dependent changes in the dimensions of T-tubular mouth regions during the cycle of contraction and relaxation may support T-tubular solute exchange with bulk extracellular fluid.^9^ At the same time, contributions of T-tubule mouth configuration to T-tubular luminal content exchange are rarely considered in experimental and computational studies.^10^

Previous studies have shown that diffusion within the T-tubular system is slower than in free solution.^11–13^ More recently, by combining fluorescent tracer measurements and detailed computational modelling we have shown that the complex T-tubular geometry gives rise to significant slowing of solute exchange, and that this slowing is more pronounced (four-fold) in mouse compared with rabbit.^10^ The diffusion slowing is a result of a combination of T-tubule tortuosity, varicosities, and presence of longitudinal elements. Additionally, slowed solute diffusion into the T-tubular system of mice, in particular, could be explained only by invoking an additional constriction to T-tubular access at the mouth region.

Here, using transmission electron microscopy (EM) and three-dimensional electron tomography (ET) we investigate the ultrastructure of T-tubule mouth regions in rat and compare it to mouse and rabbit. We find that the prominent constrictions in T-tubule mouth regions seen in mice are absent in rats, making rat T-tubule mouths structurally more similar to those in rabbit. We also provide data suggesting that T-tubular mouth structures in pig and human ventricular cardiomyocytes may be more akin to those of rabbit and rat, rather than mouse. Our findings are in keeping with the particularly slow T-tubule content exchange dynamics observed in mouse ventricular cells^10^ and they may aid improved experimental designs by careful model selection for cardiac research.

## Methods

All procedures were performed in accordance with EU legislation and with local ethical committee permission.

Cardiac tissue was obtained and ventricular myocytes were enzymatically isolated from the hearts of adult mice (C56Bl/7, ∼25 g, *N* = 7) and rabbits (New Zealand White, ∼2.5 kg, *N* = 5), as described previously.^6^ Furthermore, cardiac tissue from rats (Sprague Dawley, 180–200 g, *N* = 3) and pig (Landrace White, 45–75 kg, *N* = 1) was used. A human ventricular sample was obtained from post-mortem tissue where death was not caused by cardiovascular disease.

To optimize sample preservation, isolated cells were high-pressure frozen in buffer supplemented with 10% bovine serum albumin as cryo-protectant, using an EM PACT2 + RTS High Pressure Freezer (Leica Microsystems, Vienna, Austria). Cardiac tissue samples, in contrast, were fixed chemically, as described previously for mouse and rabbit,^10^ and for rat, pig, and human.^14^ Sarcomere lengths were comparable between all samples (1.70 ± 0.02 µm). EM and ET imaging was performed as described before.^10^^,^^15^^,^^16^

In order to investigate the ultrastructure of T-tubule mouths we used both two-dimensional EM (for qualitative overview) and three-dimensional ET data (for quantitative analyses), to ensure adequate characterisation of T-tubular mouth regions.^16^ The degree of constriction at the T-tub mouth was defined as % reduction in diameter when compared with average T-tub diameter measured across the same cell.

## Results

### Rat T-tubule openings are similar to those seen in rabbit and structurally distinct from mouse

**Figure 1:**
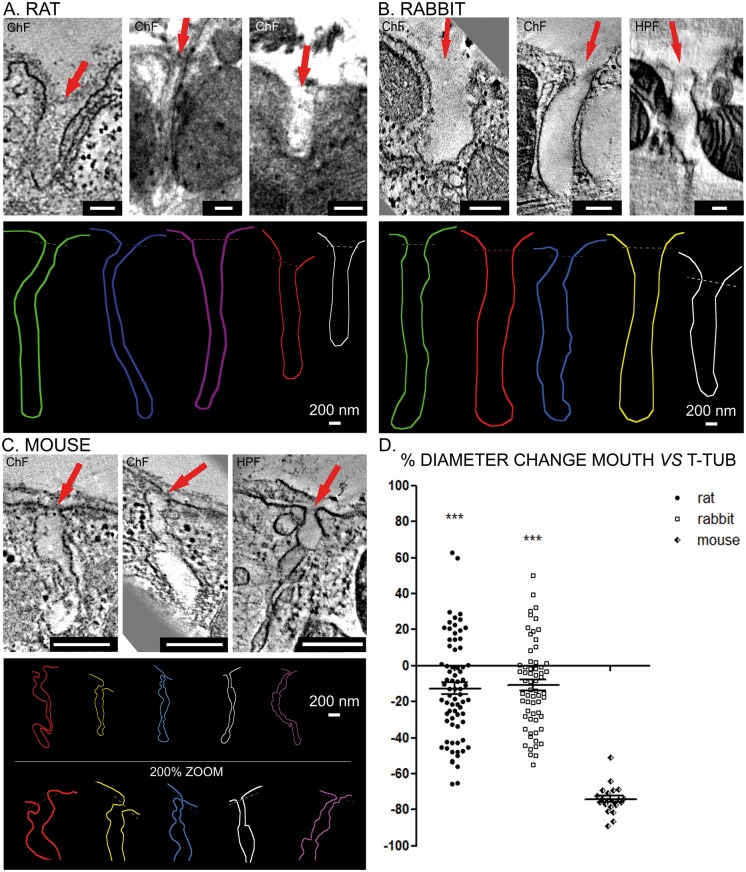
Rat ventricular T-tubule mouths do not exhibit structural constrictions and are similar to rabbit and not mouse. (*A*) Top—representative images of rat T-tubule mouths, indicated with red arrows. Bottom—representative rat T-tubule mouth rendering based on three-dimensional ET data demonstrating the structure of T-tubule openings in rat cardiomyocytes. (*B*) Top—representative images of rabbit T-tubule mouths, indicated with red arrows. Bottom—representative rabbit T-tubule mouth rendering based on three-dimensional ET data demonstrating the structure of T-tubule mouths in rabbit cardiomyocytes. (*C*) Top—representative images of mouse T-tubule mouths, indicated with red. Bottom—representative mouse T-tubule mouth rendering based on three-dimensional ET data demonstrating the structure of T-tubule mouths in mouse cardiomyocytes. (*D*) The degree of change in diameter at the mouth of the T-tubule compared with the average internal T-tubule diameter in myocytes from the same samples. Data are presented as individual points and mean ± SEM, analysed using one-way ANOVA with Bonferroni’s *post hoc* test, ***P < 0.001 vs. mouse. Mouse: *n* = 21 T-tubule mouths *N* = 7 animals; rat: *n* = 72/*N* = 3; rabbit: *n* = 64/*N* = 5. ChF, chemical fixation; ET, electron tomography; HPF, high pressure freezing; scale bars = 200 nm.

Rat T-tubule mouths have a single unobstructed lumen and are more similar to those seen in rabbits than in mouse (*Figure 1A*). The average diameter of rat T-tubule mouth regions was 205.1 ± 65.7 nm (*n* = 72 T-tubules from *N* = 3 animals). Compared with the average T-tubule diameter inside cardiomyocytes from the same samples (235 ± 71.8 nm, *n* = 70/*N* = 3; *Figure 1D*), the average constriction in the subsarcolemmal mouth region was approximately 13%. Rabbit T-tubules have equally easily traceable, single-lumen cavernous mouth regions (*Figure 1B*). The average diameter of rabbit T-tubule mouths segments was 351.7 ± 114.7 nm (*n* = 64/*N* = 5). Compared with the average diameter of T-tubules inside cardiomyocytes from the same samples (395 ± 94.2 nm, *n* = 80/*N* = 5; *Figure 1D*), the average constriction in the subsarcolemmal mouth region was around 11%.

In contrast to both rat and rabbit, in mouse ventricular myocytes, as one moves from internal T-tubules that are bounded by terminal cisternae of the SR towards the surface sarcolemma, the outermost sections of T-tubules (within ∼1μm below the surface sarcolemma) do not form a cavernous unitary space. Instead, they narrow down into a tortuous tubule of reduced diameter with local constrictions, filled with electron-dense ground substance that appears to locally occlude the tubular lumen (*Figure 1C*). The paths of these subsarcolemmal T-tubules are therefore more difficult to follow than the T-tubular system proper, even using three-dimensional ET image data volumes. The average diameter of T-tubule mouth regions in mice was 49.7 nm ±14.6 nm (*n* = 21/*N* = 7). Compared with the average T-tubular diameter inside cardiomyocytes from the same samples (185 ± 33.1 nm, *n* = 51/*N* = 7; *Figure 1D*), the average constriction of individual tube segment area by more than 73%.

### Pig and human T-tubule mouths appear similar to rat and rabbit

**Figure 2:**
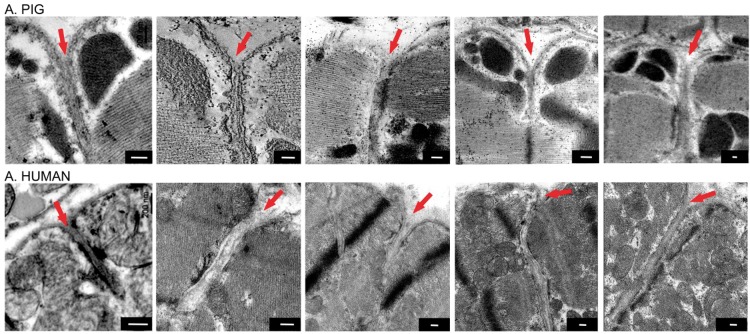
Pig and human ventricular cardiomyocyte T-tubule mouths appear qualitatively similar to those seen in rabbit and rat. Representative electron microscopic images. T-tubule mouths are indicated with red arrows. Scale bars = 200 nm.

In a pilot exploration into the qualitative appearance of ventricular T-tubule mouth regions of pig and human, we observed cavernous openings that lacked the tortuosity seen in mouse. Thus, pig (*n* = 6/*N* = 1) and human (*n* = 5/*N* = 1) T-tubule openings appear to be more similar to those seen in rat and rabbit, rather than in mouse (*Figure 2*).

## Discussion

T-tubules are highly specialized cellular structures, relevant for electrical, endocrine/paracrine and Ca^2+^‐signalling. They play central roles in excitation–contraction coupling,^17^ and the sustenance of a suitable luminal milieu is important for proper cardiomyocyte function.^18^ Here, we investigate the immediate subsarcolemmal T-tubule regions and describe species-specific differences in T-tubule mouth ultrastructure.

In all species studied, the T-tubule lumen is lined with glycocalyx,^19^ basement membrane, and interstitial ground substance of polyanionic nature,^20^ all of which may reduce the effective T-tubule diameter and restrict solute movement.^10^ T-tubule mouths in most of the species examined—rat, rabbit, pig, and human—form largely unobstructed cavernous tubes that are continuous with the deeper T-tubular system. In marked contrast, mouse T-tubules have tortuous paths in the subsarcolemmal mouth region and they show a significantly reduced (by 70%) cross-sectional area, compared with deeper T-tubules that are flanked by the SR. As ultrastructure affects T-tubular diffusion,^11^^,^^21^^,^^22^ the observed morphology may explain the previously identified restriction to solute diffusion near the access to T-tubules in mouse cardiomyocytes.^10^ As this access restriction appears to be absent from other mammalian species, it may be difficult to extrapolate data from mouse models of cardiac physiology and pathology to human.^23^ Based on the here reported data, rat, rabbit, and pig could be more suitable models to study clinically relevant T-tubule function.

The fact that the mouse, as the species with the highest heart rate studied here, and arguably, most pronounced dependence on Ca^2+^-induced Ca^2+^-release, has the largest constriction in T-tubular access appears counter-intuitive. The same could be said about average T-tubule diameters reported for murine cardiomyocytes, which tend to be only about two-thirds or less of those reported for larger mammals. It is possible that this may be related to a need to limit T-tubule solute exchange, perhaps to reduce radial solute gradients, which are increased by free(er) access to the bulk extracellular space.^10^ In connection with this point, we note that the more extensive longitudinal T-tubule elements in mice will also buffer dynamic changes in solute concentration across the myocyte. Future studies are needed to clarify these points, and to explore the relevance of species-related differences in T-tubule structure and how they change in disease.^24^

Even though diffusive access to the T-tubular system may show various restrictions in different species, none of these prevent Ca^2+^-induced Ca^2+^-release in healthy myocardium. The mouse cardiac T-tubule membrane has previously been shown to be extensively folded, a process linked to the activity of BIN1 protein.^18^ It has been proposed that such membrane folding slows diffusion within the T-tubular system and serves to maintain electrical stability of cardiomyocytes during changes in the extracellular environment, in particular during a stress response.^18^^,^^25^ However, the exact areas of the T-tubular network responsible for such restrictions in diffusion were not previously identified. The tortuous T-tubule mouth configurations shown here may form part of the explanation.^10^ Finally, the seemingly obstructed appearance of T-tubule mouths in mouse cardiomyocytes might reflect the “shedding” of vesicular BIN1-containing microparticles into extracellular space – a process thought to be responsible for BIN1 presence in circulation and linked to pathological cardiac states.^26^

## Conclusions

Future studies of cardiomyocyte electrophysiology should take into account the presence of a spatially distinct zone of reduced T-tubule cross-sectional area in mouse ventricular cardiomyocytes. This may help with the interpretation of data regarding the role of the T-tubular system in health and disease, and improve inference between species.
